# The association of PTSD symptom severity with amygdala nuclei volumes in traumatized youths

**DOI:** 10.1038/s41398-020-00974-4

**Published:** 2020-08-17

**Authors:** Olga Therese Ousdal, Anne Marita Milde, Gertrud Sofie Hafstad, Erlend Hodneland, Grete Dyb, Alexander R. Craven, Annika Melinder, Tor Endestad, Kenneth Hugdahl

**Affiliations:** 1 Department of Radiology, Haukeland University Hospital, Bergen, Norway; 2 Department of Biological and Medical Psychology, University of Bergen, Bergen, Norway; 3 NORCE Norwegian Research Centre AS, Bergen, Norway; 4 Norwegian Centre for Violence and Traumatic Stress Studies, Oslo, Norway; 5 Child and Adolescent Psychiatry Unit, Division of Mental Health and Addiction, Institute of Clinical Medicine, Faculty of Medicine, University of Oslo, Oslo, Norway; 6 NORMENT-Norwegian Center for Mental Disorders Research, University of Bergen, Bergen, Norway; 7 Department of Clinical Engineering, Haukeland University Hospital, Bergen, Norway; 8 Department of Child and Adolescent Mental Health, Oslo University hospital, Oslo, Norway; 9 Institute of Psychology, University of Oslo, Oslo, Norway; 10 Division of Neuropsychology, Helgeland Hospital, Mosjøen, Norway; 11 Division of Psychiatry, Haukeland University Hospital, Bergen, Norway

**Keywords:** Psychiatric disorders, Neuroscience

## Abstract

The amygdala is a core component in neurobiological models of stress and stress-related pathologies, including post-traumatic stress disorder (PTSD). While numerous studies have reported increased amygdala activity following traumatic stress exposure and in PTSD, the findings regarding amygdala volume have been mixed. One reason for these mixed findings may be that the amygdala has been considered as a homogenous entity, while it in fact consists of several nuclei with unique cellular and connectivity profiles. Here, we investigated amygdala nuclei volumes of the basolateral and the centrocorticomedial complex in relation to PTSD symptom severity in 47 young survivors from the 2011 Norwegian terror attack 24–36 months post-trauma. PTSD symptoms were assessed 4–5, 14–15 and 24–36 months following the trauma. We found that increased PTSD symptom severity 24–36 months post-trauma was associated with volumetric reductions of all basolateral as well as the central and the medial nuclei. However, only the lateral nucleus was associated with longitudinal symptom development, and mediated the association between 4–5 months and 24–36 months post-trauma symptoms. The results suggest that the amygdala nuclei may be differentially associated with cross-sectional and longitudinal measures of PTSD symptom severity. As such, investigations of amygdala total volume may not provide an adequate index of the association between amygdala and stress-related mental illness.

## Introduction

Experiencing an extremely traumatic event, like combat or violent assault, poses a significant threat to mental well-being. For the majority of individuals, stress reactions are transitory, however in a significant number of individuals they can endure, causing distress and mental illness, like post-traumatic stress disorder (PTSD)^1,2^. PTSD is a severe psychiatric disorder leading to tremendous personal suffering, and with current treatments being only modestly effective^3,4^. As such, identifying structural and functional brain changes associated with PTSD is of major interest, as these may yield important clues to the pathophysiology of this disease, and ultimately inform new treatments.

Current neurocircuit models of stress and PTSD focus on the amygdala^5,6^. The amygdala is an evolutionally conserved brain structure with multiple functions among which the best known is to encode and extinguish memory of fearful stimuli^5,7,8^, so as to direct physiological and behavioral responses when such stimuli are encountered. In addition to its role in fear acquisition and extinction, the amygdala plays an essential role in fear generalization^9^, arousal^10^ and processing of rewards^11^, all of which may be disrupted in PTSD. Exaggerated amygdala activity in response to trauma-related and more generic stimuli is a frequent finding in functional magnetic resonance imaging (fMRI) studies of PTSD^12,13^. The evidence from structural MRI studies are, however, inconclusive, as both volumetric increases^14^ and volumetric reductions^15^ have been reported.

One reason for the mixed findings may be that most studies have considered the amygdala as a homogenous entity, without taking its specific nuclei into account. The amygdala has historically been divided into two main complexes based on their distinct cellular architecture and connectivity^7^. The evolutionarily primitive centrocorticomedial complex (CMA), consisting of the central, medial and the cortical nuclei, is densely interconnected with the striatum, brainstem and the hypothalamus. In contrast, the evolutionarily newer basolateral complex (BLA), comprising the basal, accessory basal and the lateral nuclei, is extensively interconnected with sensory as well as prefrontal cortical areas, thalamus and the hippocampus^7^.

The results from animal models indicate distinct responses of the BLA^16,17^ and CMA^18,19^ complex to chronic or severe stress, and functional MRI studies in humans suggest that the BLA and the CMA differ in terms of activity^20^ and functional connectivity^21^ in PTSD. This renders it likely that the nuclei of the two amygdala complexes may be differently associated with PTSD. However, due to the small size of the amygdala nuclei, investigations in humans using non-invasive imaging methods have been difficult. With the recent development of automatic segmentation algorithms of amygdala nuclei and hippocampus subfields, it is now possible to look beyond overall volumetric changes and to assess specific subregions of these brain areas^22^. Of importance, these procedures yield reproducible measurements, which also correlate well with the manual delineation of amygdala nuclei and hippocampal subfields^23^. We availed ourselves of this methodology to investigate the association between long-term PTSD symptom load and amygdala nuclei volumes in 47 survivors of the 2011 Norwegian terror attack at Utøya. Rather than traditionally dividing the survivors by PTSD diagnostic status, we chose to employ a single group dimensional approach to capture a continuous spectrum of PTSD symptoms as suggested by others^24,25^. Furthermore, repeated measurements of PTSD symptom severity were obtained in 31 of the participants, thus we were also able to investigate whether PTSD symptom development or average PTSD symptom load were associated with the amygdala nuclei volumes.

## Materials and methods

### Participants

The present study was part of a larger project investigating the effects of traumatic stress on cognitive and brain measures^26,27^, and included MRI and clinical data collected from 47 survivors of the 2011 Norwegian terrorist attack at 24–36 months post-trauma. Data were collected at two sites; the University of Bergen (UiB, site 1) and the University of Oslo (UiO, site 2), Norway. Both studies were approved by the Norwegian Regional Committees for Medical and Health Research Ethics (#2012/1464 and #2011/2507) and all participants provided written informed consent before participation. For comparison purposes, we also recruited 60 age-, sex- and education-matched control subjects. General exclusion criteria were a history of neurological or severe somatic disorder, head trauma and MRI-incompatibility. In order to obtain information concerning participants’ mental status, the Mini International Neuropsychiatric Interview (MINI, 6.0.0^28^) was utilized at site 1. At site 2, all participants completed the PTSD Checklist- civilian version (PCL-C)^29^, Beck depression inventory (BDI), and the Beck anxiety inventory (BAI). Six control participants fulfilling the criteria of ongoing depression or an anxiety disorder were excluded. In addition, one person from the trauma group with incidental brain pathology discovered during the MRI exam and another trauma survivor with incomplete data were excluded. The final sample thus comprised 45 trauma survivors (mean age ± SD = 20.22 ± 2.08, 51.1% females) and 54 controls (mean age ± SD = 20.76 ± 2.71, 55.6% females).

Thirty-eight of the trauma survivors also took part in a prospective, longitudinal study on neuropsychiatric sequela of the attacks; a study lead by the Norwegian Centre for Violence and Traumatic Stress Studies (NKVTS, site 3) and approved by the Norwegian Regional Committees for Medical and Health Research Ethics (#2011/1625). Although data were collected at several time points, only data acquired 4–5 months and 14–15 months post-trauma were used in the present study. Participation included semi-structured interviews performed by professional health personnel. The interviews assessed traumatic exposures, peri-traumatic reactions, PTSD symptom scores, degree of social support, functional impairments as well as more general measures of mental health and sociodemographics^30^. PTSD symptom load was assessed using the University of California at Los Angeles PTSD Reaction Index (PTSD-RI)^30^. Combining data from the three projects was approved by the Norwegian Regional Committees for Medical and Health Research Ethics (#2017/1293).

### PTSD symptom load assessments

At site 1, PTSD symptom scores were assessed using the MINI 6.0.0^28^. The MINI is a short diagnostic structured interview that explores psychiatric diagnosis according to the DSM-IV (Axis I) and ICD-10^31^. Each question has only two response options (No = 0, Yes = 1). The PTSD diagnostic section started out by using three screening questions (i.e., H1: experienced or witnessed a significant trauma, H2: reaction to trauma and H3: re-experiencing symptoms over the last month), and if answered positively, 12 follow-up questions were asked in order to examine the presence of symptoms needed to fulfill the diagnostic criteria. PTSD load was calculated based on the number of positively answered questions for the PTSD diagnostic section (Part H in MINI 6.0.0). At site 2, subjects completed the PCL-C^29^, which is a DSM-IV based 17-item rating scale with self-report ratings ranging from 1 (not at all) to 5 (extremely) for each item. At site 3, post-traumatic stress reactions were measured using the PTSD-RI^30^. The PTSD-RI is a DSM-IV based 20-item scale in which responses are recorded on a 5-point scale, ranging from 0 (never) to 4 (most of the time). Since three of the items have two alternative formulation, only the formation leading to the highest score was utilized, resulting in 17 items being used for the total symptom scale score calculation. The PTSD symptom scores were z-standardized within each site before entering any analyses^32^.

### Imaging data acquisition and analysis

MRI data were collected 24–36 months post-trauma at sites 1 and 2. At site 1, images were acquired with a GE Signa HDx, 3 T MR scanner with an 8-channel head coil, and included a whole-brain T1 structural FSPGR sequence with a voxel size of 1 × 1 × 1 mm^3^,180 sagittal slices, TR = 7.8 ms, TE = 3.0 ms, FOV = 256 × 256 and flip angle 14°. At site 2, images were acquired with a Philips Achieva whole-body 3 T MR scanner with an 8-channel head coil, and included a whole-brain T1-weighted structural sequence with a voxel size of 1 × 1 × 1.2 mm^3^, 180 sagittal slices, TR = 6.6 ms; TE = 3.06 ms, FOV = 256 × 256 and flip angle = 8°.

**Fig. 1:**
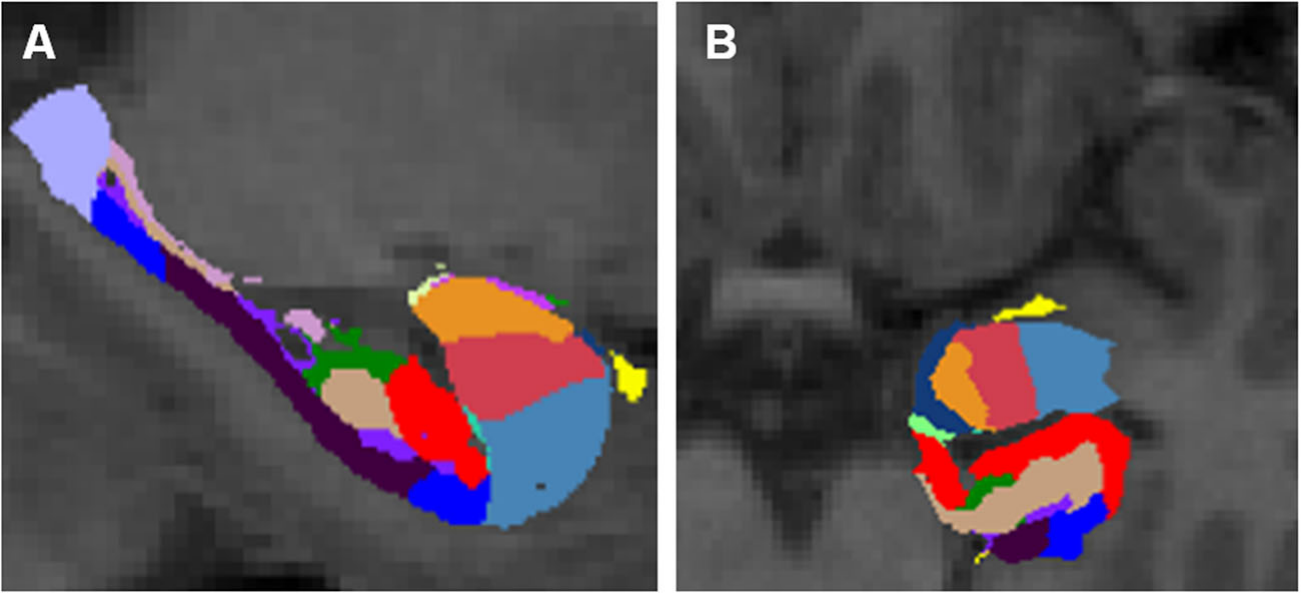
Amygdala nuclei and hippocampal subfield segmentation. The result of an amygdala nuclei and hippocampus subfield segmentation from a representative individual. A: Sagittal and B: Axial view of the color coded subfields and nuclei. Yellow: anterior amygdala area, dark blue = corticoamygdala transition zone, orange = accessory basal nucleus, pink: basal nucleus, pale blue: lateral nucleus, light green = Hippocampus amygdala transition zone; red = CA1, dark green = CA3, beige = CA4, bright blue = subiculum, dark purple = parasubiciulum, light purple = hippocampal tail, pink = fimbria.

All data were analysed within the same analysis pipeline at site 1, and were processed using FreeSurfer v6.0 (https://surfer.nmr.mgh.harvard.edu/) software, which enables fully automated volumetric segmentation of neuroanatomical structures including both bilateral hippocampus and amygdala. The segmentation procedure included the following: (a) removal of non-brain tissue using hybrid watershed/surface deformation procedures, (b) automated Talairach transformation, (c) segmentation of the subcortical white matter and deep gray matter volumetric structures, (d) tessellation of the gray/white matter boundary, (e) automated topology correction, and (f) surface deformation following intensity gradients to optimally place the gray/white and gray/CSF borders at the location where the greatest shift in intensity defines the transition to the other tissue class. After completing the fully automated-brain segmentation, we segmented bilateral amygdala into its respective nuclei, using a newly developed software extension^22^. The nuclei included the basal, the lateral, the accessory basal, the cortical, the central, the medial, the paralaminar, the corticoamygdala transition zone and the anterior amygdala area (Fig. 1a, b). All segmented data were visually inspected by a radiologist to assure the accuracy of the whole-brain segmentation. None of the subjects had to be excluded based on the visual inspection.

### Statistical analyses

Statistical analyses were performed using IBM SPSS, version 25 (IBC Corp, Armonk, New York) and R (version 3.5.0). Standardized residuals were estimated for all multiple linear regression models, and the data were reanalyzed after exclusion of all subjects with residual values >3.0 or <−3.0. Covariates that did not show at least a modest relationship with the dependent variable (*p* < 0.2) were dropped from the statistical models^33,34^. For the nuclei analyses, Bonferroni correction for the number of amygdala nuclei tested (*N* = 6, *p* = 0.008) was used to account for multiple comparisons.

Group differences in left and right total amygdala volume were tested using analyses of covariance (ANCOVA) with the amygdala volume as the dependent variable, group as fixed factor, and age, sex, site and total intracranial volume (ICV) as covariates. Next, we examined the relationship between total left and right amygdala volume and severity of PTSD symptoms 24–36 months post-trauma in the trauma survivors using multiple linear regression analyses while covarying for age, sex, ICV and site. Preliminary analyses were conducted to assess potential violations of the assumptions of normality of residuals and homoscedasticity. Moreover, we tested for multicollinearity among the independent variables. In some of the models, the assumption of homoscedasticity was not met (based on inspection of the standardized residuals plot), thus in these models, the dependent variable and the predictor of interest were log-transformed before entering any analyses. Finally, the PTSD symptom scores were z-standardized within each site^32^.

Traditionally, the amygdala has been thought of as consisting of two broad complexes, i.e. the basolateral (BLA) division and the centrocorticomedial (CMA) division^7^. While the BLA is composed of the basal, the lateral and the accessory basal nuclei, the CMA consists of the central, the medial and the cortical nuclei. To explore the impact of PTSD symptom load 24–36 months post-trauma on the nuclei of the BLA and the CMA, we performed separate multiple linear regression analyses with the amygdala nuclei volume as the dependent and PTSD symptoms, age, sex, site and ICV as the independent variables. Based on the results of the total amygdala volume analyses, only the nuclei of the right amygdala were investigated.

### Longitudinal symptom assessments

In 31 of the trauma survivors, PTSD symptom load was assessed at three time points, i.e., 4–5 months, 14–15 months and 24–36 months following the trauma. The third observation corresponded in time with the MRI scan. This gave us a unique opportunity to investigate the association between amygdala nuclei volumes and longitudinal PTSD symptom load. We first calculated the average symptom load (AUC/time) by estimating the area under the curve (AUC) and dividing this by time between the first and the last assessment:

$${\mathrm{AUC/time}} = \frac{{{\Delta}t_{01}\frac{{\left( {\mathrm{PTSD}_0 + \mathrm{PTSD}_1} \right)}}{2} + {\Delta}t_{12}\frac{{\left( {\mathrm{PTSD}_1 + \mathrm{PTSD}_2} \right)}}{2}}}{{t_{02}}}$$

where Δ*t*_01_ represents time between the 4–5 months and the 14–15 months assessments, Δ*t*_12_ represents time between the 14–15 and the 24–36 months assessments and t_02_ is the total time between the 4–5 and the 24–36 months assessments. PTSD_0_ is the PTSD symptom load 4–5 months after trauma, while PTSD_1_ and PTSD_2_ represent symptom load 14–15 and 24–36 months post-trauma, respectively. The majority of subjects experienced a symptom reduction from the first to the last assessment. By regressing each subjects’ PTSD symptom score against time point of assessment, we estimated an intercept and a linear slope, where the slope represents the individual symptom reduction from the first to the last assessment:

$${\mathrm{y}} = \beta _0 + \beta _1t$$

where *β*_0_ is the intercept, *β*_1_ is the individual linear slope and *t* is the assessments. The association between average symptom load (AUC/time) and right amygdala nuclei volumes were investigated in separate multiple linear regression models covarying for age, sex, ICV and site. Equivalent statistical models were used to test the associations between the individual symptom development (*β*_1_) and right amygdala nuclei volumes, while additionally covarying for the individual intercepts.

Based on the results from the above analyses, we finally tested if the right lateral nucleus mediated the association between PTSD symptom scores acquired 4–5 months and 24–36 months post-trauma using hierarchical linear regression as outlined in Baron and Kenny^35^. To estimate the indirect effects in the mediation model, we used the INDIRECT software as implemented in SPSS^36^. The analyses controlled for age, sex, ICV and site. Indirect effects were considered significant if the 95% confidence interval did not overlap zero^36^.

## Results

### Demographics

The demographic and clinical data of participants are presented in Table 1. Information regarding each site’s demographic and clinical characteristics are provided in Supplementary Table 1. None of the trauma survivors were prescribed any antidepressants. However, five trauma survivors occasionally used a benzodiazepine not further specified.

**Table 1:** Characteristics of the subjects.

	Trauma survivors (*N* = 45)	Controls (*N* = 54)	*p*-value
Age (mean ± SD)^a^	20.22 ± 2.08	20.76 ± 2.71	0.24
Sex (females)	23	30	0.66
Traumatic exposure (mean ± SD)^b^	0.67 ± 0.14	NA	
PTSD^c^	14	0	<0.001
Major depression^d^	9	0	0.001
Anxiety disorder^e^	17	0	<0.001

^a^Age at the time of the MRI scan.

^b^A checklist developed by NKVTS (please see^30^ for details) to assess 14 characteristics of potential traumatic exposure events (“Yes” or “No” answers). A sum (z-standardized) based on number of “yes” answers was calculated.

^c^The presence of Post-traumatic stress disorder (PTSD) was assessed by using the Mini International Neuropsychiatric Interview (M.I.N.I 6.0.0) at site 1 and the PTSD Checklist civilian version (cut-off ≥45) at site 2.

^d^The presence of a major depressive episode was assessed using M.I.N.I at site 1 and Beck Depression Inventory (BDI) (cut-off ≥18) at site 2.

^e^Site 1 utilized the M.I.N.I, and anxiety disorder refers to the presence of Generalized Anxiety disorder and/or Panic disorder. Site 2 only measured anxiety symptoms in general using the Beck Anxiety Inventory (BAI) (anxiety disorder cut-off ≥16), thus these subjects cannot be further characterized. A two-sample *t*-test was used for age comparisons between the two groups, while the *χ*^2^ test was used for sex and psychopathology comparisons.

### Total amygdala and amygdala nuclei volumes

The amygdala nuclei volumes divided by group and site are presented in Table 2. Two ANCOVAs assessing the group (i.e. trauma survivors vs. controls) difference in right (*F*_1,92_ = 0.07, *p* = 0.79, partial eta squared = 0.001) and left (*F*_1__,92_ = 1.88, *p* = 0.17, partial eta squared = 0.02) amygdala volumes while controlling for age, sex, ICV and site were not significant. A multiple linear regression analysis using the right amygdala volumes as dependent variables and the PTSD symptom load, age, sex, ICV and site as predictors, revealed a negative association between right amygdala volume and symptom load 24–36 months post-trauma (*β* = −0.34, *t* = −4.04, *p* = 0 < 0.001). An equivalent analysis for the left amygdala showed a trend towards significance (*β* = −0.17, *t* = −2.01, *p* = 0.051).

**Table 2:** Amygdala volumes divided by group and site.

	Trauma survivors				Controls			
Site	Site 1		Site 2		Site 1		Site 2	
	Mean (SD)	Range	Mean (SD)	Range	Mean (SD)	Range	Mean (SD)	Range
Left								
Lateral nucleus	651 (90)	545–957	672 (106)	493–896	659 (61)	511–837	654 (53)	567–778
Basal nucleus	454 (58)	377–622	468 (71)	332–609	457 (43)	366–545	473 (41)	400–597
Accessory basal nucleus	264 (33)	210–339	282 (44)	197–384	264 (28)	211–326	289 (28)	251–387
Central nucleus	41 (8)	25–60	47 (9)	33–63	41 (7)	28–59	47 (7)	36–69
Medial nucleus	18 (4)	11–25	21 (6)	10–34	17 (3)	11–24	21 (5)	15–33
Cortical nucleus	23 (3)	17–31	27 (5)	16–37	23 (3)	16–29	28 (4)	22–38
Right								
Lateral nucleus	699 (90)	587–934	724 (103)	521–943	691 (67)	587–867	695 (52)	606–793
Basal nucleus	490 (60)	387–661	497 (76)	342–668	474 (37)	409–583	499 (36)	418–600
Accessory basal nucleus	291 (41)	223–387	296 (46)	201–392	281 (25)	243–344	303 (22)	258–354
Central nucleus	50 (11)	28–73	51 (12)	31–76	47 (7)	31–58	51 (8)	38–67
Medial nucleus	22 (8)	11–46	22 (9)	13–50	19 (4)	11–27	22 (4)	16–34
Cortical nucleus	26 (5)	18–40	28 (5)	19–40	25 (3)	20–32	29 (3)	23–34

**Fig. 2:**
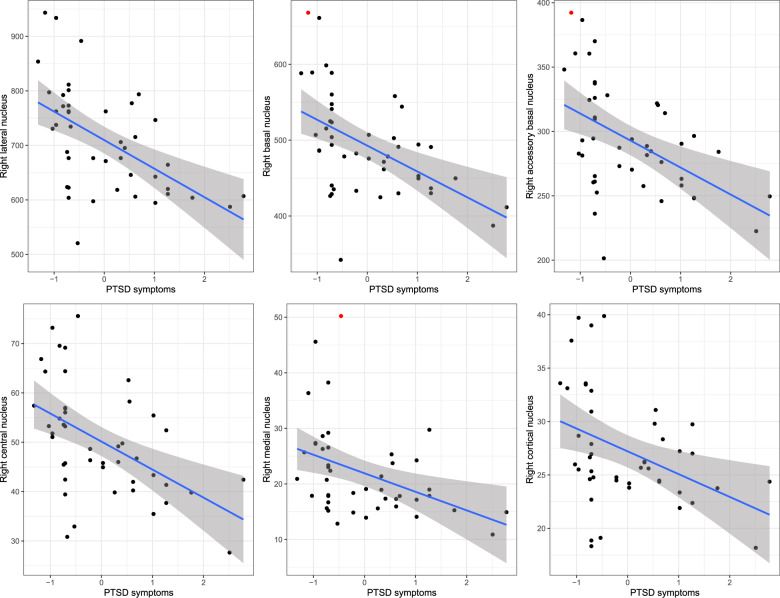
Right amygdala nuclei volumes and PTSD symptom scores. The association between the individual amygdala nuclei volumes and PTSD symptom scores (z-standardized) 24–36 months post-trauma. The regression lines represent the relationship between the dependent variable and the predictor of interest calculated without covariates. The gray shadings represent the 95% confidence interval. Outliers (residual values > 3.0 or <−3.0) are indicated by a red dot color.

Explorative multiple regression analyses of the right amygdala nuclei revealed a significant negative association between PTSD symptom load 24–36 months post-trauma and the lateral (*β* = −0.42, *t* = −3.87, *p* < 0.001, Fig. 2), the basal (*β* = −0.30, *t* = −3.63, *p* = 0.001, Fig. 2) as well as the accessory basal (*β* = −0.32, *t* = −2.95, *p* = 0.005, Fig. 2) nuclei volume when co-varying for age, sex, ICV and site. Additional negative associations emerged for the right central (*β* = −0.37, *t* = −3.75, *p* = 0.001, Fig. 2) and the right medial (*β* = −0.37, *t* = −2.90, *p* = 0.006, Fig. 2) nuclei. To further ensure that the effects were not driven by one site only, we also investigated the association between amygdala nuclei volumes and PTSD symptom load for each site separately. Additional analyses were performed to test whether the choice of PTSD symptom assessment instrument influenced our results. The results of these analyses can be found in the Supplemental Materials.

### Longitudinal PTSD symptom load

Separate multiple linear regression analyses were performed to assess the associations between average symptom load (AUC/time) and the right amygdala nuclei volumes. Although nominally significant associations emerged for the right lateral (*β* = −0.45, *t* = −2.83, *p* = 0.009), the right basal (*β* = −0.35, *t* = −2.29, *p* = 0.03) and the right central (*β* = −0.34, *t* = −2.62, *p* = 0.01) nuclei, none of these associations remained significant after correction for multiple comparisons. Next, we used the same statistical framework to assess the associations between individual symptom development (*β*_1_) and the volumes of the right amygdala nuclei. Interestingly, the analyses revealed a significant negative association for the right lateral nucleus (*β* = −0.52, *t* = −2.87, *p* = 0.008), implying that individuals experiencing less symptom reduction also had a smaller lateral nucleus volume. No other significant associations emerged after correcting for multiple comparisons (all *p*’s > 0.05).

**Fig. 3:**
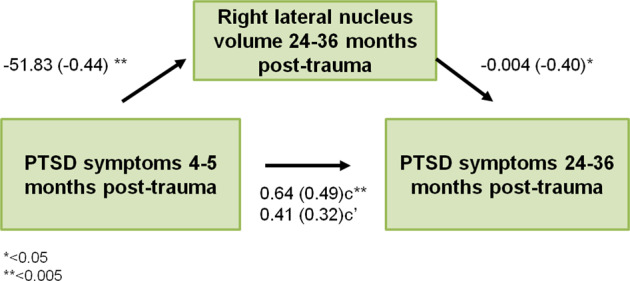
Mediation analysis. The right lateral nucleus volume mediated the relationship between PTSD symptom load acquired 4–5 and 24–36 months following the trauma. *p < 0.05, **p < 0.005. Standardized coefficients in parenthesis.

The analyses so far have demonstrated an association between the right lateral nucleus and longitudinal symptom development. As such, it is possible that post-traumatic symptoms in the early phase following a trauma may influence long-term symptoms through an impact on the lateral nucleus. To further test this hypothesis, we examined whether the right lateral nucleus mediated the association between immediate- and long-term PTSD symptom load. Using hierarchical regression, we first demonstrated that PTSD symptoms 4–5 months post-trauma predicted PTSD symptoms 24–36 months post-trauma (*B* = 0.64, *t* = 3.17, *p* = 0.003, Fig. 3). A second regression showed that the PTSD symptoms 4–5 months post-trauma was associated with right lateral nucleus volume 24–36 months post-trauma (*B* = −51,83, *t* = −3.12, *p* = 0.004, Fig. 3). Right lateral nucleus volume was also associated with concurrent PTSD symptoms (i.e. 24–36 months following trauma) (*B* = −0.004, *t* = −2.18, *p* = 0.04, Fig. 3). Importantly, adding the right lateral nucleus volume as a second predictor for PTSD symptom load 24–36 after trauma moderated the effect of PTSD symptom load 4–5 months post-trauma (*B* = 0.41, *t* = 1.88, *p* = 0.07, Fig. 3), and the indirect effect of the right lateral nucleus volume on long-term PTSD symptoms was significant (bootstrap results for indirect effect; 95% CI [0.03, 0.57]), consistent with a mediating role.

## Discussion

We provide converging evidence of long-term effects of a traumatic event during adolescence on amygdala volume. More specifically, traumatized youths showed reduction of amygdala volume with increase in PTSD symptom severity 24–36 months post-trauma, which is in line with previous studies in PTSD patients^25,32^. Perhaps more interestingly, the subdivision analyses revealed that the negative association between amygdala volume and symptom severity could be ascribed to the nuclei of the BLA complex as well as the right central and medial nuclei. However, only the lateral nucleus was associated with individual PTSD symptom development, and mediated the association between short- and long-term PTSD symptoms. The results indicate that the various amygdala nuclei may be differentially associated with cross-sectional and longitudinal measures of PTSD symptom load. Future studies may therefore benefit from considering the amygdala as a heterogeneous brain area, when understanding the relationship between amygdala structure and PTSD.

One possible explanation for the conflicting amygdala volumetric findings in PTSD may be that previous studies have treated the amygdala as a homogeneous entity, and not taken its structural and functional heterogeneity into account^25^. The nuclei of the BLA and the CMA have unique cellular architectures and structural connections^37^, which is reflected in their distinct roles in fear learning- and regulation^8,38^. In line with this notion, the volume of the individual amygdala nuclei may be uniquely affected in disorders altering fear sensation^39^. Moreover, whereas increased spinogenesis and dendritic growth of principal and stellate neurons have been reported in the BLA following severe stress^16,17^, a loss of stellate neuron spines may occur in the CMA nuclei^18,19^. Finally, preliminary findings from human functional imaging studies suggest that the BLA and the CMA differ in terms of activity^20^ and functional connectivity^21^ in PTSD, further suggesting that these complexes should be considered separately in trauma- and stress-related disorders^40,41^.

We here report a negative association between long-term (i.e. 24–36 months) PTSD symptom severity and the nuclei of the BLA complex. The results are corroborated by findings of unique structural alterations of the BLA in animals exposed to repeated restraint stress^40,41^. Although stress-dependent structural changes in animals are mainly trophic^40,41^, it has been suggested that the initial volumetric expansion may be followed by a long-term volumetric reduction in humans^42^. This is plausible, given that the BLA contains abundant glucocorticoid receptors^41^, and thus stress and excessive amounts of glucocorticoids may have direct and indirect neurotoxic effects on the BLA complex, inhibiting dendritic expansion and even causing neuronal loss. Furthermore, other stress-related mental illnesses like depression are also associated with initial amygdala volumetric increases^43^ followed by volumetric reductions upon recurrent depressive episodes^44^. Of note, rodents with smaller BLA show stronger conditioned fear responses and corticosteroid responses to stress^45^, and humans with a genetically rare disease (Urbach-Wiethe) damaging the BLA show increased vigilance in response to threat cues^46^. As such, the increased arousal and vigilance, which is part of the PTSD symptom complex may be at least partially mediated by structural changes in BLA. This is further suggested by an inverse relationship between total amygdala volume and amygdala activity^47^, providing a link between our findings and the more frequently reported amygdala hyperactivity in PTSD^13^.

We also found evidence for an association between long-term PTSD symptom severity and concurrent volumes of the central and the medial nuclei. A recent study using vertex-based neuroimaging identified specific abnormalities in the morphology of the CMA which scaled with PTSD load^24^. In addition, a study in young PTSD patients found altered gray matter density and intrinsic connectivity of both the BLA and CMA complexes^48^. The central nucleus of the CMA is essential for fear expression and autonomic arousal in response to threat cues, and receives numerous connections from the lateral and basal nuclei^7^. Interestingly, the communication between the lateral and the central nucleus is regulated by prefrontal inputs^49^. As such, aberrant medial prefrontal—BLA connectivity in PTSD patients^21^ may facilitate signaling through the lateral—central nuclei route, with potential consequences for the central nucleus structure. Nevertheless, a combined effect on both the BLA and CMA could explain why PTSD is likely to affect both fear learning and expression, and also why extinguishing fear is so difficult in this disorder^50,51^.

We had the unique opportunity to investigate the association between PTSD symptom severity acquired at several time-points (i.e. 4–5, 14–15 and 24–36 months) post-trauma and long-term amygdala nuclei volumes. Interestingly, we found that the individual PTSD symptom development was closely related to the lateral nucleus volume 24–36 months post-trauma. Moreover, the right lateral nucleus volume mediated the association between short- and long-term PTSD symptoms. The findings are in line with a recent study showing that amygdala reactivity immediately following the index trauma is related to PTSD symptoms months post-trauma^6^. In addition, previous studies have reported that amygdala reactivity to affective stimuli pre-deployment positively predicted post-deployment PTSD symptoms in military samples^52,53^, and that post-traumatic stress symptoms in the aftermath of an index trauma were negatively associated with total amygdala volume 24 years later^54^. The present study extends these findings by showing that the long-term lateral nucleus volume is associated with early symptom development, and indeed may mediate the association between short-and long-term outcome. As such, nuclei of the BLA may be an essential target of early interventions including pharmacological or psychological treatments following trauma, to prevent the development of chronic PTSD.

Although our study may add novel insight into the association between amygdala volume and PTSD, several questions remain unanswered. One important question relates to whether lower amygdala nuclei volumes are a consequence of the extreme stress exposure per se or represent a preexisting vulnerability for developing PTSD. Findings of altered amygdala morphology in animals exposed to stress^16–19^ as well as altered structure and function in humans exposed to early life adversity^55,56^ may suggest an effect of stress per se. In contrast, the observation of reduced amygdala volume in PTSD patients relative to trauma-exposed control subjects without PTSD^15^ is comparable with the hypothesis that lower amygdala volume is a heritable risk factor for developing or a consequence of having PTSD. However, to directly answer this question would require research studies using prospective, longitudinal designs and twin studies.

We acknowledge that a potential limitation of the present study rests in the heterogeneity related to the trauma group. This is always likely to be a challenge in these types of studies given the variability of response to stressors. A second limitation is the use of different MRI scanners and PTSD symptom assessment instruments which may have influenced the results. In addition to the use of different PTSD instruments, the majority of subjects had PTSD symptom scores in the lower ranges of the continuum. It is not clear whether the findings would be similar if more subjects with increased PTSD severity had been recruited. Therefore, future potential replication studies should be conducted in larger samples with a more even distribution of subjects across PTSD symptom severity. Although PTSD symptoms were assessed at multiple time-points, none of the subjects were assessed prior to the trauma, and thus the study cannot disentangle pre-existing aberrations from trauma-induced changes. Furthermore, traumatic experiences was not an exclusion criteria for the control subjects, which may have influenced the group comparison (i.e. trauma survivors vs controls). Moreover, our analyses were quite selective, as only the right lateral nucleus was subjected to a mediation analysis. Finally, we acknowledge that the FreeSurfer v6.0 extension used to segment the amygdala nuclei is a developmental version, and thus the results warrant replication in future samples with a greater diversity of PTSD symptom severity.

The present findings indicate that long-term PTSD symptom severity in the aftermath of trauma is associated with concurrent volumetric reduction of all basolateral nuclei as well as the central and the medial nuclei. However, only the lateral nucleus volume predicted the individual longitudinal PTSD symptom development and mediated the association between 4–5 months and 24–36 months PTSD symptom load. Our findings suggest that the amygdala nuclei may be differentially associated with cross-sectional and longitudinal measures of PTSD symptom severity. Accordingly, total amygdala volume alone may not provide a reliable index of the association between amygdala and stress-related mental illness.

## Supplementary information

Supplemental Material_Amygdala_nuclei_volumes_in_traumatized_youths
