# Supplementary material for: The association of PTSD symptom severity with amygdala nuclei volumes in traumatized youths
Source: Transl Psychiatry. 2020 Aug 17;10:288. doi: 10.1038/s41398-020-00974-4 (PMC7431855; doi:10.1038/s41398-020-00974-4)
Supplement: Supplementary file 1 — Supplemental Material_Amygdala_nuclei_volumes_in_traumatized_youths [file 41398_2020_974_MOESM1_ESM.docx]

# The association of PTSD symptom severity with amygdala nuclei volumes in traumatized youths

# Supplemental Materials

## Supplementary Results

**Amygdala nuclei volumes and PTSD symptom load separated by site**

In site specific multiple linear regression models, we tested the association between total right or left amygdala volume and PTSD symptom load, while covarying for ICV. Preliminary analyses were conducted to ensure no violation of the assumptions of normality of residuals, multicollinearity and homoscedasticity. The analyses revealed significant negative associations between symptom load and right amygdala volumes at both sites (site 1: β=-0.46, t= -3.90, p=0.001 site 2: β=-0.35, t=-2.12, p<0.05), but only at site 1 for the left amygdala (site 1: β=-0.29, t= -2.17, p=0.04, site 2: β=-0.21, t=-1.29, p=0.21). Although both sites showed negative associations between PTSD symptom load and volumes of the basolateral as well as the medial and the central nuclei, the associations were generally stronger at site 1 (lateral nucleus site 1: β=-0.48, t= -3.66, p=0.001, lateral nucleus site 2: β=-0.37, t= -2.03, p=0.06, basal nucleus site 1: β=-0.44, t= -3.67, p=0.001, basal nucleus site 2: β=-0.30, t= -1.83, p=0.08, accessory basal nucleus site 1: β=-0.40, t= -2.71, p=0.01, accessory basal nucleus site 2: β=-0.30, t= -1.92, p=0.07, medial nucleus site 1: β=-0.37, t= -2.41, p=0.03, medial nucleus site 2: β=-0.20, t= -0.97, p=0.35, central nucleus site 1: β=-0.48, t= -3.69, p=0.001, central nucleus site 2: β=-0.25, t= -1.52, p=0.15).

**Amygdala nuclei volumes and longitudinal PTSD symptom load using binarized PCL-C scores**

To further ensure that our results were not driven by the use of different PTSD symptom assessments, we also binarized the score on each item of the PCL-C (i.e. symptom present=1, symptom not present=0), to harmonize with the M.I.N.I. The resultant sum score was used in multiple regression analyses to assess the association between PTSD symptom severity 24-36 months post-trauma and right amygdala nuclei volumes while covarying for site, ICV, age and sex. In line with previous results, the analyses revealed significant associations between all BLA nuclei (right lateral nucleus: β =-0.51, t=-3.92, p<0.001, right basal nucleus: β =-0.38, t=-3.85, p<0.001, right ABB: β =-0.34, t=-3.04, p=0.004) and longterm PTSD symptom severity. For the CMA nuclei, only the association between the central nucleus and longterm PTSD symptom severity was replicated (right central nucleus β =-0.43, t=-3.59, p=0.001).

**Amygdala volume and the factor analytic structure of PTSD**

In addition to the heterogeneous nature of the amygdala, PTSD may also have a heterogeneous phenotypic expression. Several studies have confirmed that PTSD may be represented by symptom clusters, like the four factor model of Simms et al[^1^](#_ENREF_1). We here tested for an association between the four different symptom clusters, i.e. re-experiencing, avoidance, dysphoria and arousal[^1^](#_ENREF_1) and the volume of right amygdala using general linear models covarying for ICV. The analyses were run separately for each site. Bonferroni correction for the number of factors tested (N=4, p=0.12) was used to account for multiple comparisons. Although nominal significant associations emerged at site 1 (re-experiencing: β =-0.32, t=-2.35, p=0.03, avoidance: β =-0.29, t=-2.11, p<0.05, dysphoria: β =-0.33, t=-2.44,p=,0.02 arousal: β =-0.34, t=-2.57,p=0.02) and site 2 (arousal: β =-0.38, t=-2.21, p=0.04) none of these survived correction for multiple comparisons.

# References

1. Simms LJ, Watson D, Doebbeling BN. Confirmatory factor analyses of posttraumatic stress symptoms in deployed and nondeployed veterans of the Gulf War. *Journal of abnormal psychology* **111,** 637-647(2002).

# Supplementary Table 1: Clinical and demographic information for the trauma survivors and the controls separated by site

| **Site** | **N** | | **Age (mean ± SD)** | | **Sex (females)** | | **PTSD^1^ (rate)** | | **PTSD^1^ (symptom range)** | | **MD^2^ (rate)** | | **MD^2^ (symptom range)** | | **Anxiety disorder^3^ (rate)** | | **Anxiety disorder^3^ (symptom range)** | |
| --- | --- | --- | --- | --- | --- | --- | --- | --- | --- | --- | --- | --- | --- | --- | --- | --- | --- | --- |
|  | **T** | **C** | **T** | **C** | **T** | **C** | **T** | **C** | **T** | **C** | **T** | **C** | **T** | **C** | **T** | **C** | **T** | **C** |
| 1 | 24 | 30 | 19.8±1.6 | 20.3±2.3 | 15 | 17 | 7 | 0 | 1-15 | 0-9 | 3 | 0 | 0-8 | 0-2 | 8 | 0 | ^4^0-9  ^5^0-17 | ^4^0  ^5^0-7 |
| 2 | 21 | 24 | 20.8±2.4 | 21.4±3.0 | 8 | 13 | 7 | 0 | 21-78 | 17-30 | 6 | 0 | 3-37 | 0-9 | 9 | 0 | 3-45 | 0-13 |

^1^The rate and symptom range of Post-traumatic stress disorder (PTSD) were assessed using the Mini International Neuropsychiatric Interview (M.I.N.I 6.0.0) at site 1 and the PTSD Checklist civilian version (PTSD cutoff≥45) at site 2.^2^ The rate and symptom range of an ongoing major depressive episode were assessed using (M.I.N.I) at site 1 and Beck Depression Inventory (BDI) (depressive episode cutoff ≥18) at site 2. ^3^Site 1 utilized the M.I.N.I, thus this category includes Generalized anxiety disorder and Panic disorder. The symptom ranges (^4^ Generalized anxiety disorder symptom range, ^5^ Panic disorder symptom range) are presented separately, while the rate includes both disorders. Site 2 assessed anxiety symptoms with the Beck Anxiety Inventory (BAI) (anxiety disorder cutoff ≥ 16), thus these subjects cannot be further characterized. Abbreviations: T=Trauma survivors, C=Controls, PTSD=Post-traumatic stress disorder, MD=Major Depression (ongoing)

^rate and symptom range of Post-updated accordinglyucleus mediates the association between 4-5 and 24-36 months PTSD symptom loa^
